# The Moloch of Civilized Man

**Published:** 1907-08

**Authors:** 


### EDITORIAL DEPARTMENT.

### THE MOLOCH OF CIVILIZED MAN.

The fire-god of the Ammonites in Canaan, to whom human sacrifices were fed, was not more voracious than is the demon "Drink" of our boasted civilization. The former was fed by fanaticism, the latter "for revenue only"; the one by the priests of Baal, the latter by an enlightened government for pay. For pay the government licenses certain executioners; licenses them to slowly poison the young and the old; to make widows and orphans; to make murderers, thieves, prostitutes and paupers; to make lunatics, who transmit the poison to the fourth generation, when the race or family becomes extinct. Meantime, the State must care for the victims in costly asylums at an ever-increasing expense; care for the paupers and prosecute the criminals—the cost of all which largely exceeds the revenue. Is this not monstrous? Is it not time that a cry went up all over the land for protection against this death and destruction-dealing Meloch, *drink*? In Austin at a recent election twelve hundred mothers, wives, sisters and daughters pleaded for protection of their loved ones—all in vain. But it will come! As sure as fate—the saloon will go. An advancing civilization and every consideration of public safety, social economies, morals and religion, of race integrity, demand it. Hasten the day, oh, righteous God!

Bad enough it would be were the individual only sacrificed. But the drunkard—even the "moderate drinker"—hands down the accursed thirst to his children's children. A child has rights, even prenatal. A child has the right to be well born, and not to "be cast into this breathing world scarce half made up," as the horrible Gloster said. He should not be handicapped with a feeble body and a weak mind, an inheritance from a drinking father.

It has been shown time and again, and a thousand times over, that alcohol is the arch enemy of the human race, and yet—and yet—"local option," they say, "kills business." I deny it. But if there is any community whose "business" will be killed by temperance, any town that can not flourish and prosper without the accursed saloon, that town should suffer the fate of Gomorrah of old, and be damned!

In the June *Red Back* it was shown on the highest authority, and the authority named, that in Texas in 1860 there were sixty insane people in a population of about 600,000, and there was one asylum, to build and support which cost $12,000, or one insane to 12,080 inhabitants.

From 1860 to 1904 the population increased 504 per cent, while the insane increased 6800 per cent, or 13.7 faster than the population. That is, for every unit added by birth or immigration, there were added 13.7 insane.

At present Texas has four asylums with 4500 inmates, and there are 500 insane in jail,—there being no room for them in the asylums. That is, there is now one lunatic to every 600 population, and the cost of maintenance of these asylums for the past five years has been over $3,500,000, or $707,000 a year average.

It was shown by the superintendents of these asylums that over half of the insane (one says 95 per cent—one 60) were insane because of alcohol, direct or by inheritance. It was shown that the total revenue to the State from liquor licenses falls short by $107,000 annually of being sufficient to care for the insane, and far short of paying for the enforcement of the criminal laws,—over 50 per cent of the criminals being made criminal by drink or inheritance of the drink mania.

The editorial in June attracted the attention of Dr. C. L. Gregory, the newly appointed Superintendent of the North Texas Insane Asylum at Terrell, and he wrote me the following letter with permission to publish it, sending at the same time the letters referred to.

I have summarized those letters briefly, and I present them herewith as additional proof, if any be needed, that the demon Alcohol is destroying the people of the United States at a fearful rate, and hand in hand with one of its other victims—tuberculosis, in which it is a large factor,—is compassing the sure decadence of the American people. Call me a crank, call me an alarmist, if you will, but answer the *facts* given above, and, taking them as a basis of calculation, extend the time into the future and answer these questions: By the time (say in twenty years) Texas gets the 5,000,000 population that the "club" is working for—how many insane will there be in Texas; how many asylums will be required to care for them, and what will their maintenance cost the taxpayers? Why not stop the production of insane? Remove the cause?

Dr. Gregory writes as follows:

NORTH TEXAS
HOSPITAL FOR THE INSANE.

TERRELL, TEXAS, July 6, 1907.

*Dr. F. E. Daniel, Austin, Texas.*

DEAR DOCTOR: I have just received a copy of the "Red Back," and read with much interest and pleasure the article styled "Cause and Effect." I desire to congratulate you upon the noble fight you are making against alcohol as a factor in the production of insanity.

I beg to hand you check for the subscription of the "Red Back," and ask that you record me as a lifetime reader.

The malignant role played by alcohol in the production of insanity and other nervous diseases is appalling, but the baleful effects of alcohol are not expended and confined to the generation addicted to its use, but by destroying the integrity of nerve structures and lowering the standard of organic relations, the progeny is thus affected; and we harvest weak, nervous impairment, epilepsy and insanity. Failure to teach and warn the children of the danger along this line is fraught with direst effects,—suffering to both children and parents, and the element of moral and successful training should claim our most earnest consideration. If children learn to shun this grave danger in youth, it will be a fixed principle of their life, and their will power will be sufficiently strong to resist these evils, and control the depraved tendencies, keeping them under absolute subjection, which will lead the will powers into higher and nobler channels. If these laws are not observed utter ruin and depravity will come to the untrained boy when he comes in contact with the population of life.

I place at your disposal a number of letters received by me from asylum superintendents. Sincerely yours,

CHAS. S. GREGORY, M. D.,
Superintendent North Texas Hospital for the Insane.

The Earl of Shaftsbury, who was at the head of the English Lunacy Commission fifty years, says that in his opinion fully 50 per cent of all insanity is caused by alcohol.—Morris S. Guth, M. D., Superintendent State Hospital for Insane, Warren, Pa.

*Illinois.*—Illinois Western Hospital for the Insane, Watertown, Ills., Dr. W. E. Taylor, Superintendent. Of those (inmates) we are certain of, I estimate approximately 30 per cent of the men are insane because of alcohol.

*Michigan.*—Hospital for the Insane, Newbury, Mich., Dr. E. H. Campbell, Superintendent. Thirty-two per cent of our patients have a history of excessive use of alcohol. Fourteen per cent are true alcoholic psychoses.

*Missouri.*—St. Louis Insane Asylum, 1906-7. Of total males, 174—74 were excessive drinkers; 52 regular drinkers; 11 dipsomaniacs; 137, or 78.7 per cent. Females, 142—17 were excessive drinkers; 18 regular drinkers; 3 dipsomaniacs; 38, or 25 per cent. Total, male and female, 316; insane from alcohol, 175, or 55.5 per cent male and female.

*New York.*—Utica State Hospital, Dr. H. L. Palmer, Superintendent. Insanity resulted directly or indirectly from alcohol for the past two years 11.28 per cent. In men alone, 19.3 per cent.

Rochester State Hospital, Dr. E. H. Howard, superintendent. Analysis of two years, 502 cases, 239 men, 263 women.—In 48 per cent of men and 10 per cent of women, alcoholic etiology.

*Massachusetts.*—Danvers Insane Hospital, Hawthorn, Mass. Dr. C. W. Page, Superintendent. For several years.—The percentage amongst male patients that can be traced directly to alcohol varies from 18 to 20 of annual admissions of 250 to 300. This does not include the large number of cases one or both of whose parents have been intemperate. These figures are cases of insanity arising directly from alcohol.

Fredericksburg State Hospital.—Dr. Jno. H. Nichols, Superintendent. Of 687 patients in hospital October 1, 1906, in about 25 per cent insanity was caused by alcohol.

Medfield Insane Asylum, Harding, Mass., Dr. Edw. French, Superintendent. About 27 per cent are alcoholics.

Worcester Insane Asylum, Worcester, Mass., Dr. E. V. Scribner, Superintendent. At least one-third of all cases have become insane either from direct or indirect effects of alcohol.

*Vermont.*—Battleboro Retreat, Battleboro, Vt., Dr. S. E. Scott, Superintendent. Insanity inmates caused by alcohol: Males, 35 per cent; females, 5.

*Pennsylvania.*—State Hospital for Insane, Norristown, Pa., Dr. W. W. Richardson, Superintendent. At least 20 to 25 per cent of cases can be assigned to this cause (alcohol).

State Hospital, Warren, Pa., Dr. M. S. Guth, Superintendent. Probably 25 per cent. "In so many cases is it associated with other causes, such as heredity, that it is difficult to decide whether it is a primary or secondary cause."

*New Hampshire.*—State Hospital, Concord, N. H., Dr. C. P. Bancroft, Superintendent. "In a general way, I should say that so far as it is ascertained, fully 35 per cent among male admissions are referable to alcohol, and 40 per cent to heredity (alcoholic). Families and individuals oftentimes conceal facts."

*Nebraska.*—State Hospital, Ingleside, Neb., Dr. W. B. Kern, Superintendent.

In over 30 per cent of patients admitted, alcohol either directly or indirectly is responsible for mental state.

*California.*—State Hospital, Stockton, Cal., Dr. Fred P. Clark, Superintendent. Nearly 16 per cent of total number (insane from alcohol).

*Washington.*—Eastern Washington Hospital for the Insane, Medical Lake, Washington, Dr. W. H. Anderson, Superintendent. About 40 per cent of the male and 5 per cent of female patients are insane from the effects of alcohol directly or indirectly.

*West Virginia.*—Second Hospital for Insane, Spencer, W. Va., Dr. A. J. Lyons, Superintendent. Of 250 male patients, about 15 per cent have been committed as a result of the use of narcotics ("whisky, cigarettes, etc.").

*Kansas.*—Topeka State Hospital, Topeka Kan., Dr. T. C. Biddle, Superintendent. Insanity in 35 per cent of our male patients * * * was caused by it (alcohol and narcotics).

*Colorado.*—Woodcroft Hospital, Pueblo, Colo., Dr. Hubert Work, Superintendent. (Insanity caused by) "Alcoholic drinks, 42 per cent in males; 7 per cent in females."

*Wisconsin.*—Milwaukee Sanitarium, Wauwatosa, Wis., Dr. Richard Dewey, Superintendent. About 16 per cent, due directly or indirectly to alcohol.

*Texas.*—Austin Insane Asylum, Dr. B. M. Worsham, Superintendent. Twenty per cent due directly to alcohol; 75 per cent to heredity.

Southwest Texas Insane Asylum, San Antonio, Dr. M. L. Graves, Superintendent. In TEXAS MEDICAL JOURNAL, April, 1905, says: "The important factors in the production of insanity are heredity (alcoholism) in its various forms,"—and estimates the percentage at 60.

*The saloon must go!* It is demanded by every consideration of humanity, public morals and safety and even race integrity! Listen to the cry of the widows and orphans and the ravings of the lunatic and the condemned murderer and ask yourself, Mr. Whisky Advocate, if you are not *particeps criminis?*

